# Biodegradation of MC252 oil in oil:sand aggregates in a coastal headland beach environment

**DOI:** 10.3389/fmicb.2014.00161

**Published:** 2014-04-10

**Authors:** Vijaikrishnah Elango, Marilany Urbano, Kendall R. Lemelle, John H. Pardue

**Affiliations:** Department of Civil and Environmental Engineering, Louisiana State UniversityBaton Rouge, LA, USA

**Keywords:** PAHs, crude oil, beach, *Deepwater Horizon*, biodegradation, alkanes, biogeochemistry

## Abstract

Unique oil:sand aggregates, termed surface residue balls (SRBs), were formed on coastal headland beaches along the northern Gulf of Mexico as emulsified MC252 crude oil mixed with sand following the *Deepwater Horizon* spill event. The objective of this study is to assess the biodegradation potential of crude oil components in these aggregates using multiple lines of evidence on a heavily-impacted coastal headland beach in Louisiana, USA. SRBs were sampled over a 19-month period on the supratidal beach environment with reasonable control over and knowledge of the residence time of the aggregates on the beach surface. Polycyclic aromatic hydrocarbons (PAHs) and alkane concentration ratios were measured including PAH/C30-hopane, C2/C3 phenanthrenes, C2/C3 dibenzothiophenes and alkane/C30-hopane and demonstrated that biodegradation was occurring in SRBs in the supratidal. These biodegradation reactions occurred over time frames relevant to the coastal processes moving SRBs off the beach. In contrast, submerged oil mat samples from the intertidal did not demonstrate chemical changes consistent with biodegradation. Review and analysis of additional biogeochemical parameters suggested the existence of a moisture and nutrient-limited biodegradation regime on the supratidal beach environment. At this location, SRBs possess moisture contents <2% and molar C:N ratios from 131–323, well outside of optimal values for biodegradation in the literature. Despite these limitations, biodegradation of PAHs and alkanes proceeded at relevant rates (2–8 year^−1^) due in part to the presence of degrading populations, i.e., *Mycobacterium sp*., adapted to these conditions. For submerged oil mat samples in the intertidal, an oxygen and salinity-impacted regime is proposed that severely limits biodegradation of alkanes and PAHs in this environment. These results support the hypothesis that SRBs deposited at different locations on the beach have different biogeochemical characteristics (e.g., moisture, salinity, terminal electron acceptors, nutrient, and oil composition) due, in part, to their location on the landscape.

## Introduction

MC252 oil reaching the shoreline from the *Deepwater Horizon* blowout was primarily in the form of a water-in-oil emulsion. As these emulsions reached sandy beach shorelines, they mixed with sand and shell to produce several unique oil forms including thick agglomerated deposits, termed oil “mats” or “tarmats,” and smaller oil:sand aggregates, termed “surface residue balls” or SRBs. SRBs are typically 0.5–5 cm in diameter (Urbano et al., 2013). The aggregates are stable in that they can be easily handled without breaking and can be transported by waves and currents across the beach. SRBs are often imprecisely referred to as “tar balls” which are solid or semi-solid pieces of weathered oil which wash onto beaches worldwide from natural and anthropogenic sources (Nemirovskaya, 2011; Suneel et al., 2013). Characterization of some of the biogeochemical properties of SRBs has been performed for risk and fate purposes (OSAT-II, 2011; Urbano et al., 2013), but longer-term weathering or biodegradation studies are limited (Aeppli et al., 2012; Hall et al., 2013).

The presence of soil aggregates can inhibit the rate and extent of biodegradation processes of hydrocarbons by a number of mechanisms including pore size exclusion of microbial populations and diffusion limitations on supplies of key nutrients and electron acceptors (Monrozier et al., 1991; Scow and Alexander, 1992; Nocentini and Pinelli, 2001; Nam et al., 2003). The rate of biodegradation is often inversely related to aggregate size (Scow and Alexander, 1992). The SRB aggregates generally have higher porosities (Urbano et al., 2013) than clay aggregates that may mitigate diffusion limitations. However, SRB aggregate structure can provide protection from predation for hydrocarbon-degrading microbial populations and limit desiccation in moisture-limited environments. Larger aggregate sizes with their larger pore structure can create opportunities for biostimulation, i.e., addition of nutrients and oxygen (Chang et al., 2013).

Rate and extent of microbial degradation of crude oil components can be heavily influenced by geochemical parameters including temperature (Mohn and Stewart, 2000; Eriksson et al., 2001; Haritash and Kaushik, 2009), salinity (Kastner et al., 1998; Diaz et al., 2002; Badejo et al., 2013), nutrient content (Dibble and Bartha, 1979; Chen et al., 2008; Tejeda-Agredano et al., 2011) and the availability of electron acceptors such as oxygen (Tang et al., 2006; Uribe-Jongbloed and Bishop, 2007; Haritash and Kaushik, 2009; Ortega-Calvo and Gschwend, 2010). On beach environments, additions of nutrients and organic matter have enhanced biodegradation rates of MC252 oil (Horel et al., 2012; Mortazavi et al., 2013) confirming previous studies (Bragg et al., 1994; Xu and Obbard, 2003). Background levels of nutrients are important in demonstrating a positive effect for fertilization on crude oil degrading consortia on beaches (Venosa et al., 1996). For crude oil classes of n-alkanes and polycyclic aromatic hydrocarbons (PAHs), optimal geochemical conditions for biodegradation are typically aerobic, low to moderate salinity conditions with sufficient available nitrogen and phosphorus to approximate cellular molar ratios of C:N:P (total carbon:total nitrogen:total phosphorus) of 100:10:1. The goal of this study was to determine and assess the biogeochemical conditions contributing to biodegradation of crude oil components in these coastal systems.

This study focuses on the a heavily impacted coastal headland beach system, the Caminada Headlands, which consists of 2 segments, Elmer's Island and Fourchon Beach. This study will couple new oil composition and biogeochemical data with previous measurements on SRBs (Urbano et al., 2013) to make the case for the importance of biodegradation as an important fate process for these aggregates on the coastal headland beach environment. These systems are extremely dynamic, with tropical storms and strong cold fronts reworking the sand. The regularity of these storm events means that surface SRBs are washed away over time frames of months to years. Therefore, weathering reactions need to occur over time scales less than storm-driven transport to be relevant to hydrocarbon fate. Chemical analyses conducted for a suite of alkanes and PAHs on hundreds of SRBs and oil mat samples will be coupled with data on biogeochemical parameters to establish evidence for biodegradation potential. This data will also be used to develop hypotheses on which biogeochemical conditions that may be limiting the rate and extent of biodegradation of crude oil components in these aggregates. This paper provides the biodegradation potential assessment for a larger study on beach fate that has included papers on SRB characterization (Urbano et al., 2013) and the statistical distribution of SRBs on the beach surface (Lemelle et al., 2014).

## Methods and materials

### Field sampling

**Figure 1:**
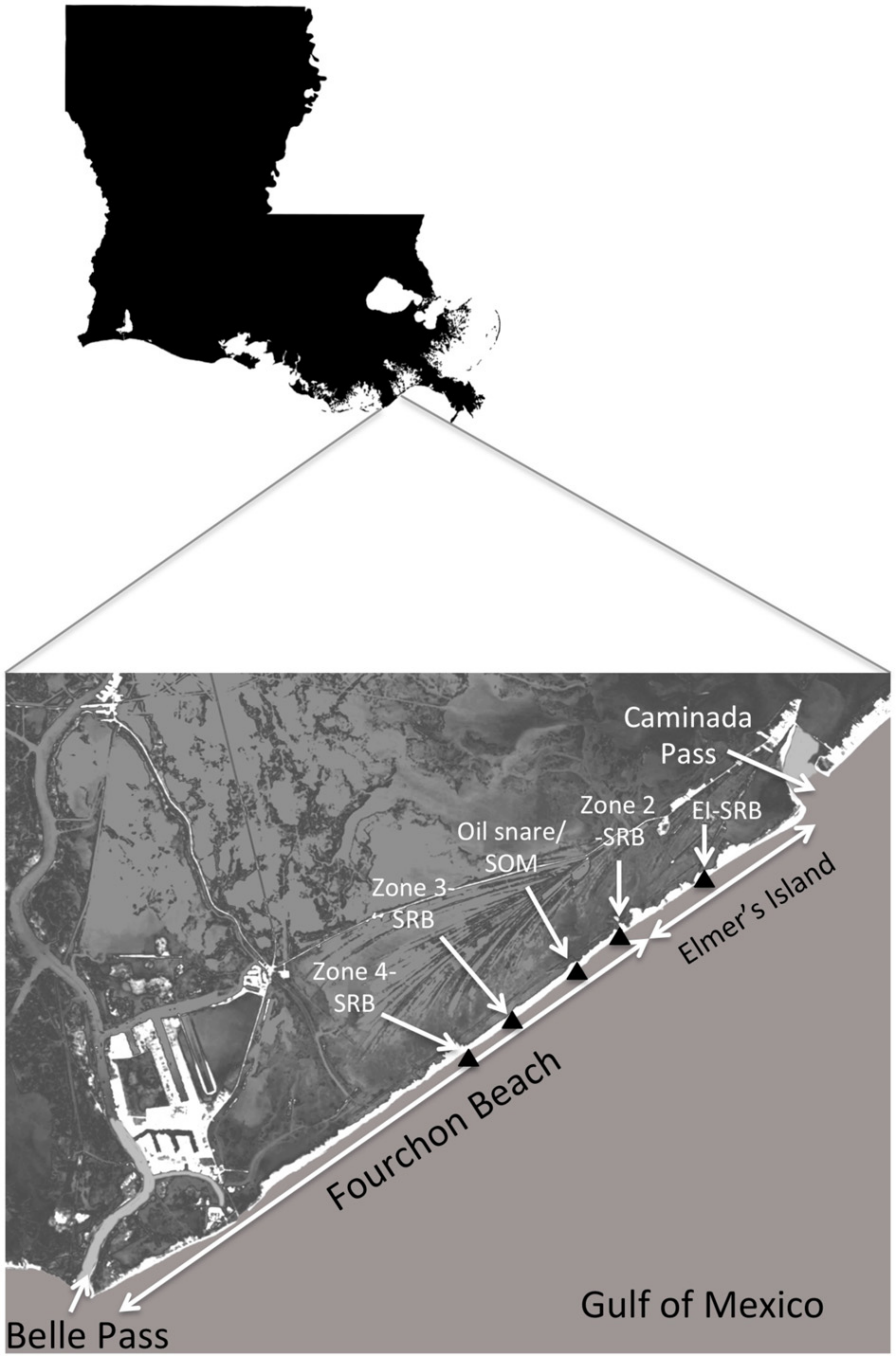
**Map of sampling locations on Fourchon Beach and Elmer's Island, Louisiana**.

SRBs were sampled from 5 distinct areas on the supratidal portion of the Caminada Headlands beach, 4 sites on Fourchon Beach and 1 site on Elmer's Island (Figure 1). Oil first reached this shoreline beginning on May 20, 2010. Two of the sites on Fourchon Beach, termed the “Zone 3” site and the “Zone 4” site were set aside from clean-up activities from October 2010 through June 2011. The sites were located north of the beach crest in the supratidal zone. SRBs were deposited on these beach segments by 2 tropical events, Hurricane Alex and TS Bonnie, which brought high tide and storm surge to the beach in June and July of 2010, respectively. The 2 sites were inspected daily and were unaffected by high tides, storm surge or cleanup activities during the sampling period: for the Zone 4 site (10/26/10–11/11/2010) and for the Zone 3 site (12/15/11–12/21/11). The Zone 3 site was sampled again for SRBs on 5/18/11 after inspection and tidal records revealed that tidal surge did not impact this site from December 2010 to May 2011. SRBs were completely washed off of both sites during TS Lee, whose storm surge impacted the beach in September 2011.

The Zone 3 and Zone 4 sites each consisted of an area 30.5 m × 30.5 m with surface coverage of SRBs ranging from 0.01 to 8.1%. Both sites were sampled using randomized block methods without resampling. Briefly, 0.9 m × 0.9 m sampling areas were selected randomly, photographed, and 2 individual SRBs collected from the surface. In addition, composite SRB samples were obtained by sampling and sieving the top 5 cm of each selected area. Zone 4 was sampled on 10/26/2010 (day 159 since oil reached the shoreline), 11/04/2010 (day 167) and 11/11/2010 (day 174). Zone 3 was sampled on 12/15/2010 (day 208), 12/21/2010 (day 214) and 05/18/2011 (day 362). Information on the statistical surface and size distribution of SRBs is presented in a separate paper (Lemelle et al., 2014).

A third SRB location on Fourchon Beach (N 29°09.34', W 090°06.38') was sampled on 8/4/2011 (day 439) at an area east of the previous sampling areas in Zone 2. These SRBs were part of a broader characterization study of the biogeochemical characteristics of the oil:sand aggregates (Urbano et al., 2013). These SRBs were presumed to have reached the supratidal during the same storms as the other 2 Fourchon sites and therefore extend the time frame of weathering an additional 2.5 months from the last samples taken from the Zone 3 site. Like the Zone 3 and Zone 4 sites, SRBs on the surface were completely removed by storm surge during TS Lee in 9/2011.

The fourth sampling location was on the supratidal of Elmer's Island, a segment of the Caminada Headlands beach east of Fourchon Beach. This location had a deposition field of SRBs on the edge of sand dunes at a slightly higher elevation than the beach surface. Sampling details are described in Urbano et al. (2013). These SRBs were not mobilized during TS Lee storm surge in 2011 and therefore allowed sampling of SRBs to continue through May 2012. Representative SRBs were obtained on 10/20/2011 (day 547), 2/9/2012 (day 628) and 5/31/2012 (day 739). Storm surge from Hurricane Isaac in August 2012 removed the remaining SRBs from this location. In total, SRB samples were obtained sequentially from 10/26/10 through 5/31/2012.

A fifth set of samples was obtained immediately after TS Lee in September 2011. These consisted of 2 sample types: submerged oil mat (SOM) samples and oil associated with oil snare, a pom-pom type of oil adsorbent used extensively on these beach during the active spill response. SOMs are larger oil, sand and shell agglomerations that were buried on the beach surface or offshore as a result of storm events described above. TS Lee broke up the subtidal mat and samples were obtained from the beach surface. Snare oil samples were associated with a set of pom-pom adsorbent left behind on the beach during the response and uncovered during TS Lee. These samples are significant since the oil snare was buried in the upper intertidal, where regular inundation of seawater would occur, but without complete submergence. Biogeochemical characteristics of these samples are described in Urbano et al. (2013).

MC252 oil was confirmed using the form of the oil as the identifying feature. The study focused solely on SRBs and SOMs; the oil, sand and shell aggregates that formed as emulsified MC252 mixed with sand and shell in the nearshore environment. These oil forms are unique to this spill in the Caminada Headlands environment. Condensed oil forms from other sources, including tar balls and tar patties, were occasionally observed at much lower frequencies to SRBs and SOMs. However, tar forms are visibly distinct by color, shape, and texture and therefore, were not sampled during the activities described above.

### PAH and n-Alkane analysis

The SRBs, SOM and oil snare samples were extracted and analyzed for PAH, alkanes and hopane biomarkers. Discrete SRBs, SOM, and oil snare samples were extracted without drying. Composite samples from Zones 3 and 4 sampling sites were dried in a greenhouse for 5–7 days and sieved (30 USA standard testing sieve with 0.60 mm nominal opening) in the laboratory to separate SRBs from the beach sand. Subsamples of approximately 10 g were placed in a 50 mL Teflon tube with 20 mL of acetone/hexane (50:50 v/v) mixture and tumbled at room temperature for 48 h. Repetitive extraction on selected samples demonstrated less than 10% of oil remains after this step. The tumbled Teflon tubes were centrifuged (Beckman Coulter Avanti J-20 XPI) at 8000 rpm for 10 min and the upper solvent phase in the tube was removed. The solvent was dried over Na_2_SO_4_ and further concentrated to a 10 mL volume with a RapidVap N2 evaporation system (Labconco). The solvent extract was analyzed by injecting 1 μL onto a Hewlett Packard 6890N gas chromatograph equipped with HP 6890 series autosampler, DB 5 capillary column (30 m × 0.25 mm × 0.25 μm film) and HP 5973 mass selective detector. The temperature program for analysis was: injector and detector at 300°C and 280°C respectively and the oven temperature program used was: 45°C for 3 min, increased at 6°C/min to 315°C and hold for 15 min. Helium at 5.7 mL/min was used as the carrier gas. Quantitation was done in selected ion monitoring mode using internal standards after calibration with alkane, alkylated PAH and biomarker standards. Daily quality control included blanks and continuing calibration standards for analytes. Precision of the combined extraction and analytical method was within 15% relative standard deviation (RSD), based on replicate analyses.

The following PAHs were quantified: naphthalene (NAPH), C1-naphthalenes (C1-NAPH), C2-naphthalenes (C2-NAPH), acenaphthylene (ACENAPH), acenaphthene (ACE), fluorene (FLU), C3-naphthalenes (C3-NAPH), phenanthrene (PHEN), C1-phenanthrenes (C1-PHEN), C2-fluorenes (C2-FLU), C1-dibenzothiophenes (C1-DiBENZ), fluoranthene (FLUOR), pyrene, C2-phenanthrenes (C2-PHEN), C3-fluorenes (C3-FLU), C2-dibenzothiophenes (C2-DiBENZ), C1-pyrene/fluoranthene, C3-phenanthrenes (C3-PHEN), C3-dibenzothiophenes (C3-DiBENZ), chrysene (CHRYS), C4-phenanthrenes (C4-PHEN), C1-chrysenes (C1-CHRYS), C2-chrysenes (C2-CHRYS), and C3-chrysenes (C3-CHRYS). Total PAHs were computed as the sum of the detected compounds in this list.

The following alkanes were analyzed: decane (C10), undecane (C11), tridecane (C13), tetradecane (C14), pentadecane (C15), hexadecane (C16), heptadecane (C17), pristane, octadecane (C18), n-eicosane (C20), docosane (C22), n-tetracosane (C24), n-hexacosane (C26), n-octacosane (C28), n-tricontane (C30), n-dotricontane (C32), and n-hexatriacontane (C36). 17α(H),21β (H)-hopane (30αβ), herafter referred to as C30-hopane, was also quantified.

### Nutrient and moisture content analysis

Total C and N were measured on intact SRBs, SOM, and snare oil pieces. Total carbon was measured via combustion and coulometric detection using a modified ASTM D5373 method. Nitrogen was determined on a Thermo Flash EA 1112 analyzer. The N technique utilized was the classical Dumas method, using thermal conductivity detection. The method is described in ASTM D5373 (coal) and ASTM D5291 (petroleum products). Moisture content was determined by loss on drying overnight at 105°C. The moisture content determination did not distinguish between loss of moisture and volatile losses from oil at these temperatures. The absence of alkanes below C15 in these weathered oil samples demonstrates the low volatile content of the oil, however.

### Statistical analysis and rate computation

All statistical analyses were done using one and two tailed student *t*-test at 95% confidence interval. First-order rate constants from declines in PAHs and alkanes were calculated using nonlinear regression of data pooled from all the samples from each sampling event.

## Results

### Chemical evidence for SRB biodegradation potential

**Figure 2:**
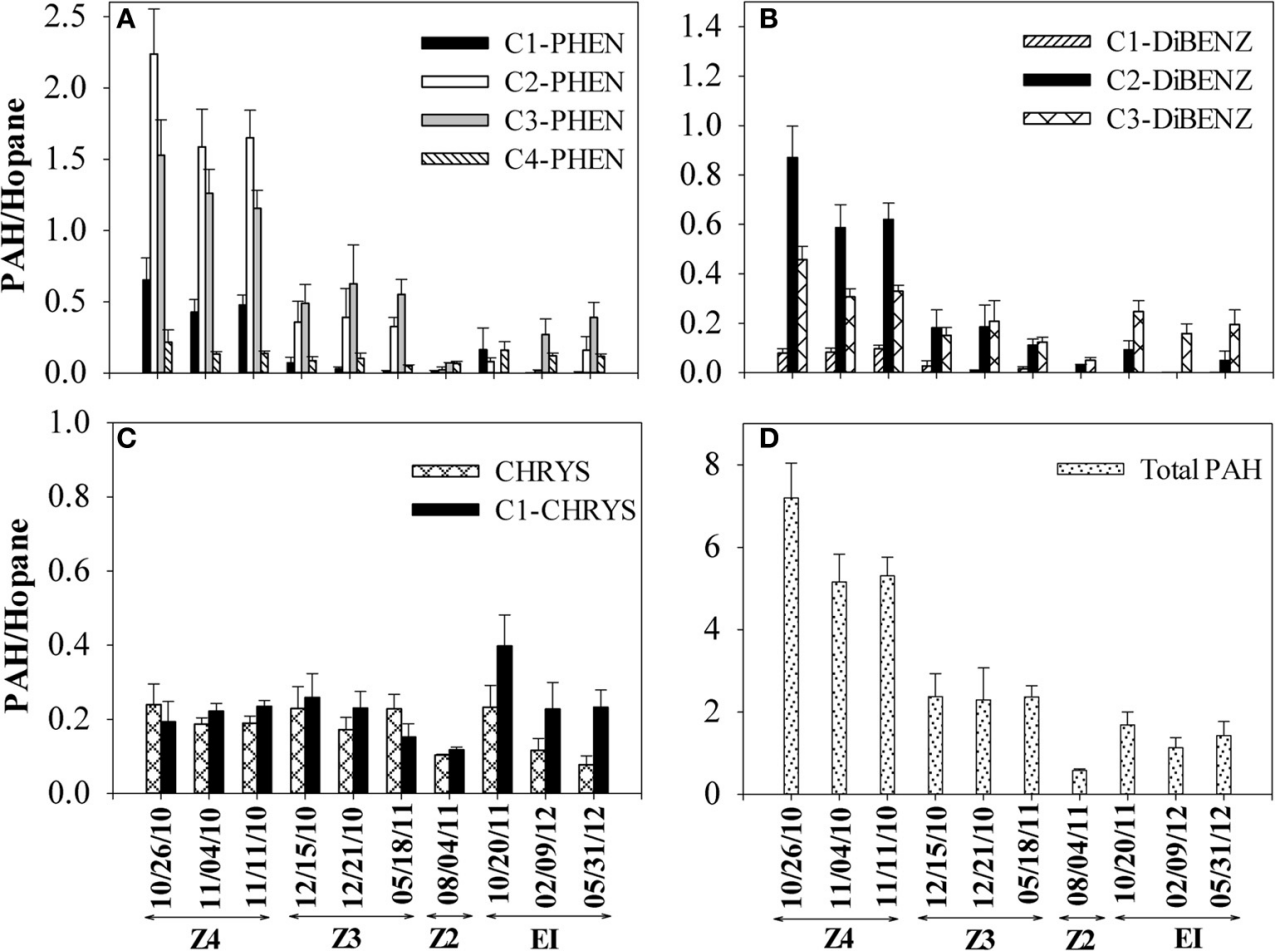
**Changes in PAH/hopane ratios in SRBs from 4 sampling locations [Fourchon Beach (FB) Zone 4, FB Zone 3, FB Zone 2, and Elmer's Island (EI)] sampled over time**. **(A)** C1–C4 phenanthrenes **(B)** C1–C3 dibenzothiophenes, **(C)** chrysene and C1-chrysenes and **(D)** total PAHs. Dates correspond to the following times since oil reached the shoreline beginning on May 20, 2010: 10/26/2010 (day 159), 11/04/2010 (day 167), 11/11/2010 (day 174), 12/15/2010 (day 208), 12/21/2010 (day 214), 05/18/2011 (day 362), 8/4/2011 (day 439), 10/20/2011 (day 547), 2/9/2012 (day 628) and 5/31/2012 (day 739).

Concentrations of PAHs and other crude oil components were used to compute ratios that tracked changes in more biodegradable compounds to less biodegradable compounds over time. Two sets of ratios were computed for the aromatic fraction: comparing PAHs to the poorly biodegradable biomarker C30-hopane (Prince et al., 1994) and comparing concentrations of C2-phenanthrenes and C2-dibenzothiophenes to their more alkylated homologues, C3-phenanthrenes and C3-dibenzothiophenes (Michel and Hayes, 1999). These ratios were computed for SRBs on the Caminada Headlands beach over a 19-month interval. Declines in PAHs relative to C30-hopane were observed in the 4 sampling locations on Fourchon Beach and Elmer's Island (Figure 2). Total PAH/hopane ratios declined from 7 to 0.5 over this time frame (Figure 2D). The dominant PAHs observed were C1-, C2-, C3- and C4-phenanthrenes, C1-, C2- and C3-dibenzothiophenes, chrysene, and C1-chrysene (Figure 2). The lower molecular weight naphthalenes, acenaphthylene, acenaphthene, fluorene, alkylated fluorenes, unsubtituted phenanthrene, and dibenzothiophene were observed at minor levels or below our detection limit of 0.05 mg/kg. By the last sampling event in May 2012, the greatest percentage decrease was observed for C1- and C2-phenanthrenes, followed by C2- and C3-dibenzothiophenes and C3- and C4-phenanthrenes. No apparent change in chrysene and C1-chrysene were observed when the data was normalized to C30-hopane (Figure 2C). This is consistent with the lower rate and extent of biodegradation of 4-ring PAHs like chrysenes (Haritash and Kaushik, 2009).

**Figure 3:**
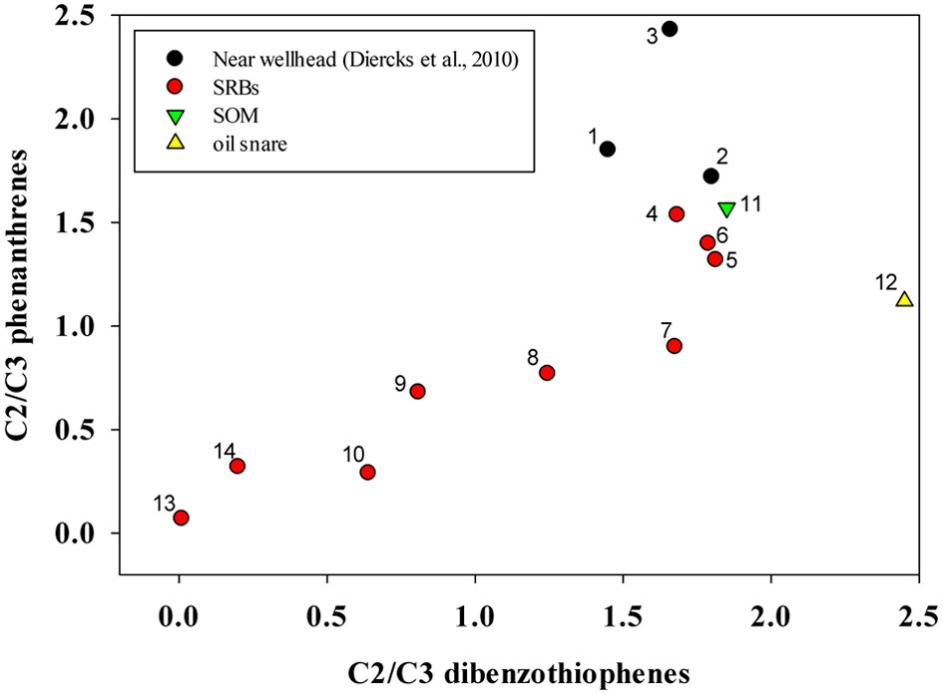
**Changes in C2/C3 phenanthrenes and C2/C3 dibenzo thiophenes in SRB, SOM and oil snare samples compared with oil samples from near the wellhead**. For SRB, SOM, and oil snare samples, points represent means of multiple discrete samples taken on individual sampling dates. Samples are numbered chronologically by sampling date. 1. Sea surface, site 30 5/10/10 (Diercks et al., 2010), 2. 1320 m, Site 33, 5/12/10 (Diercks et al., 2010), 3. 1160 m, Site 34, 5/12/10 (Diercks et al., 2010), 4. Fourchon Beach (FB) 10/26/2010, *n* = 7, 5. FB 11/4/2010, *n* = 20, 6. FB 11/11/2010, *n* = 24, 7. FB 12/15/2010, *n* = 20, 8. FB 12/21/2010, *n* = 17, 9. FB 5/18/2011, *n* = 17, 10. FB 08/04/11, *n* = 2, 11. FB SOM 09/08/11, *n* = 9, 12. FB Oil Snare 09/08/11, *n* = 10, 13. Elmer's Island (EI) 02/09/2012, *n* = 9, 14. EI 05/31/2012, *n* = 15.

Ratios of C2/C3 phenanthrenes and C2/C3 dibenzothiophenes were computed and compared using a “double ratio” plot that can illustrate temporal changes in PAH composition (Figure 3) (Michel and Hayes, 1999). For comparison, the plot includes ratios computed from open ocean samples from near the wellhead taken during the week of 05/09/2010 (Diercks et al., 2010) to contrast the magnitude of the immediate weathering after the spill. SRB datapoints on Figure 3 represent averages of SRBs sampled in this study from the 4 study sites described above. They show a clear trend of movement toward the origin over time, which occurs as the concentration of the C2 homolog declines with respect to the C3 homolog. This is consistent with biodegradation patterns of these alkylated 3 ring PAHs, not due to physical weathering reactions such as dispersion or volatilization (Wang et al., 1998). One other trends is apparent from this double-ratio plot is the change in the C2/C3 phenanthrenes ratio from samples at sea and onto the Fourchon Beach shoreline without a corresponding change in the ratio of C2–C3-dibenzothiophenes (Figure 3). Subsequent changes in both ratios in the SRBs on the beach did not show any bias toward phenanthrenes or dibenzothiophenes and both ratios declined approximately equally.

**Figure 4:**
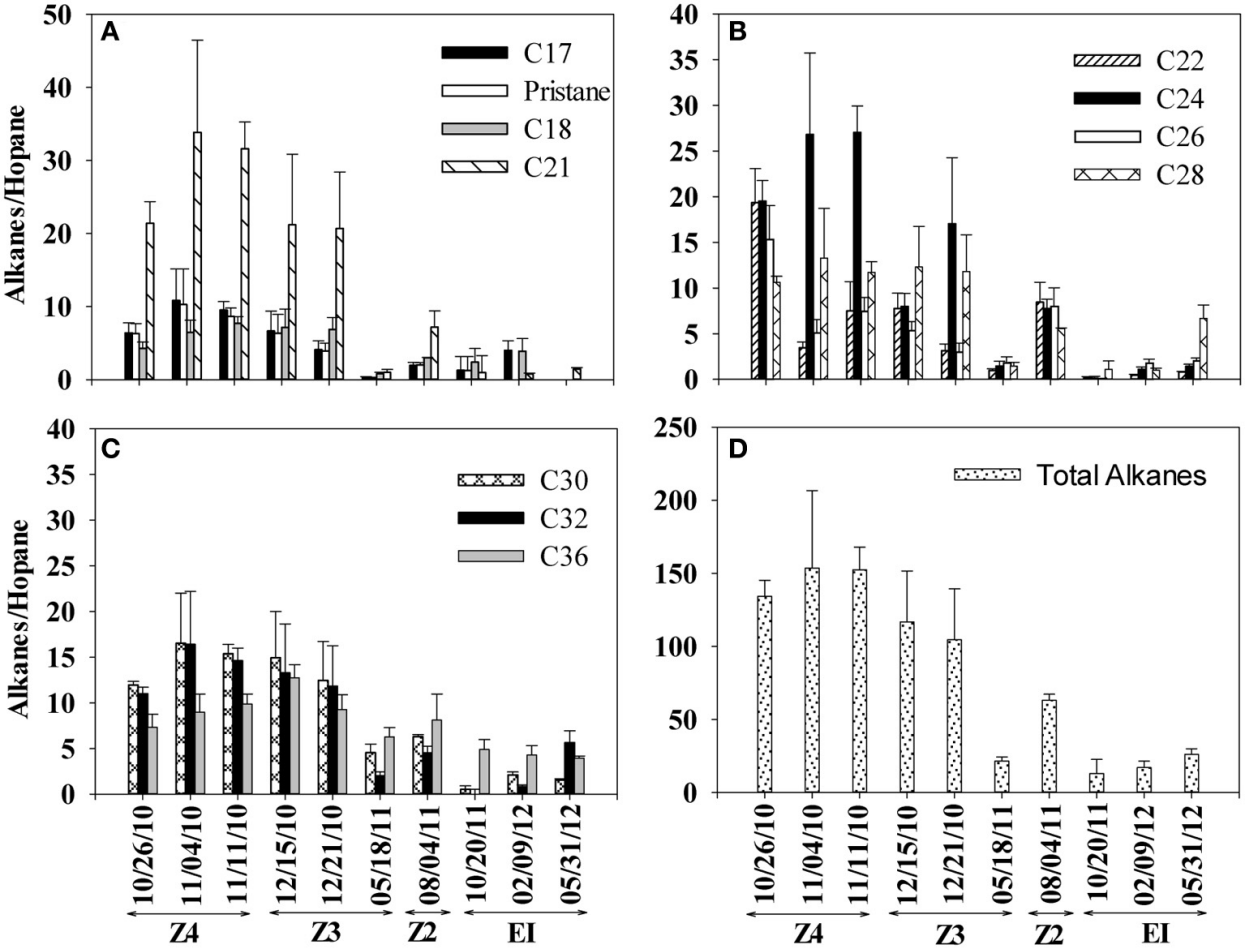
**Changes in alkane/hopane ratios in SRBs from 4 sampling locations [Fourchon Beach (FB) Zone 4, FB Zone 3, FB Zone 2, and Elmer's Island (EI)] sampled over time**. **(A)** C17, pristane, C18, and C21 **(B)** C22, C24, C26, and C28 **(C)** C30, C32, and C36 **(D)** total alkanes. Dates correspond to the following times since oil reached the shoreline beginning on May 20, 2010: 10/26/2010 (day 159), 11/04/2010 (day 167), 11/11/2010 (day 174), 12/15/2010 (day 208), 12/21/2010 (day 214), 05/18/2011 (day 362), 8/4/2011 (day 439), 10/20/2011 (day 547), 2/9/2012 (day 628) and 5/31/2012 (day 739).

Similar to PAHs, we observed a consistent decrease in alkane/hopane ratios from October 2010 to May 2011 (Figure 4). Between October 2010 and May 2011, the total alkane ratio decreased by approximately 85% (Figure 4D). Alkane/hopane ratios did not decline appreciably in the later Elmer's Island samples and at the end of the sampling period in May 2012, measurable alkanes were still observed in the SRBs. Among alkanes (Figure 4), only C17–C36 alkanes were observed consistently in all of the SRB samples. Lighter alkanes C10–C12 were below our detection limit for all the samples. In total, alkane concentrations were roughly 10x those of the PAHs.

A second set of biodegradation ratios were computed, the ratios of C26–C15, C16 and C17 (Table 1). These ratios have also be used to evaluate loss due to biodegradation (Hazen et al., 2010) on the principle that lower molecular weight alkanes should biodegrade faster than higher molecular weight alkanes. Initially, these ratios (C26/C15, C26/C16, and C26/C17) were two to three orders of magnitude higher than what was observed in MC252 oil and other ocean samples collected immediately after the spill (Hazen et al., 2010) (Table 1). The ratio of C26–C15 consistently decreased during our sampling period and the difference between October 2010 and May 2011 was statistically significant (*p* = 0.003). For the C26/C16 ratio, differences observed between October 2010 and May 2011 sampling events were not statistically significant (*p* = 0.91). However, C15 and C16 alkanes were close to our detection limit in all our samples and only minor losses were observed during our sampling period. Therefore, the decrease in the ratio was primarily due to decreases observed in C26. The difference in C26/C17 ratio was also not significant between October 2010 and May 2011 sampling events (*p* = 0.67). Elevated ratios in Table 1 suggest the persistence of C26 relative to C15, C16, and C17, which is consistent with other field observations where long chain alkanes were more persistent over short chain alkanes (Venosa et al., 1996; Hazen et al., 2010; Liu et al., 2012) and where slow biodegradation of long chain alkanes was observed in oiled sands (Rodriguez-Blanco et al., 2010; Kostka et al., 2011). Ultimately, two trends from the alkane data in Figure 4 and Table 1 stand out. These are the persistence of some alkanes in the SRBs over the length of the sampling period and the variability in the alkane results between SRBs, which results in the high observed standard deviations in Table 1.

**Table 1:** **Ratio of C26 to C15, C16 and C17 in SRBs from Fourchon Beach (FB) and Elmer's Island (EI)**.

**Sample ID**	**Date**	**N**	**C26/C15**	**C26/C16**	**C26/C17**
FB Zone 4	10/26/2010	7	260 ± 79	86 ± 52	9.8 ± 7.1
FB Zone 4	11/4/2010	20	120 ± 52	170 ± 100	4.7 ± 2.8
FB Zone 4	11/11/2010	24	150 ± 31	93 ± 50	5.6 ± 3.1
FB Zone 3	12/15/2010	20	67 ± 10	41 ± 21	4.5 ± 0.57
FB Zone 3	12/21/2010	17	57 ± 11	85 ± 11	3.8 ± 0.99
FB Zone 3	5/18/2011	17	40 ± 30	75 ± 57	12 ± 1.7
FB Zone 2	08/04/11	2	bdl^a^	bdl	bdl
EI SRB	10/20/11	9	bdl	bdl	bdl
EI SRB	02/09/2012	9	bdl	bdl	bdl
EI SRB	05/31/2012	15	bdl	bdl	bdl
MC252^b^	5/2010		1.61	0.85	0.56
BM057104^b,c^	5/2010		3.4	1.8	1.1
BM058104^b,c^	5/2010		2.84	1.48	0.8

a *bdl = samples below detection for C15, C16, or C17*.

b *Hazen et al., 2010*.

c *BM057104 and BM058104 are samples collected 10 km away from wellhead*.

### Rate constants for PAHs and alkanes in SRBs

First-order rate constants (year^−1^) for declines in PAH and alkane concentrations in the SRBs are of the same order of magnitude for most compounds (Table 2). However, the initial concentrations of alkanes in SRBs are an order of magnitude higher than PAHs and therefore, would persist longer in the environment. C1-phenanthrenes, the lowest molecular weight PAHs evaluated for this study, had the highest loss rate and the more alkylated phenanthrenes (C2, C3, and C4) were 48, 25, and 39% lower respectively. Among dibenzothiophenes, C1-dibenzothiophene had the highest loss rate but was only 58% of that for C1-phenanthrene. Chrysene and C1-chrysenes weathering rates were the lowest of all the PAHs quantitated in this study consistent with the persistence of heavier molecular weight PAHs. In order to account for the variability observed in the standard error, statistical analysis were performed to determine if the rates were significantly different. The loss rates were statistically different for C3-phenanthrenes (*p* = 0.044), C2-dibenzothiophenes (*p* = 0.048), chrysene (*p* = 0.012) and C1-chrysenes (*p* = 0.016), when compared with C1-phenanthrene. However, the loss rates were not statistically different for C2-phenanthrene (*p* = 0.16), C4-phenanthrene (*p* = 0.08), C1-dibenzothiphene (*p* = 0.13), C3-dibenzothiophene when compared to C1-phenanthrene. The ratio of rates of C2/C3 phenanthrenes (1.9) was not statistically different from the ratio of rates of C2/C3 dibenzothiophenes (1.72), which explains the nearly equal change in these ratios in SRBs in Figure 3.

**Table 2:** **First-order rate constants for declines in PAH and alkane concentrations from Fourchon Beach and Elmer's Island from 2010–2012**.

**PAH**	**Rate (1/year)**	**Alkanes**	**Rate (1/year)**
C1-PHEN	8.0 ± 2.4^a^	C17	6.4 ± 0.24
C2-PHEN	3.8 ± 1.3	C18	3.4 ± 0.68
C3-PHEN	2.0 ± 0.78	C21	6.3 ± 0.38
C4-PHEN	3.1 ± 0.59	C22	6.0 ± 1.4
C1-DiBENZ	4.6 ± 2.5	C24	5.6 ± 0.58
C2-DiBENZ	2.2 ± 0.68	C26	4.6 ± 1.2
C3-DiBENZE	3.8 ± 0.79	C28	3.8 ± 0.48
CHRYS	0.28 ± 0.33	C30	2.3 ± 0.15
C1-CHRYS	0.76 ± 0.19	C32	3.8 ± 0.25
Total PAHs	3.05 ± 0.49	C36	0.46 ± 0.39
		Total alkanes	3.6 ± 0.15

a *±standard error*.

Among alkanes, the rate constants were highest for C17 (6.4 year^−1^) and, while rate constants for C21, C22, C24, and C26 were lower by 2–29%, they were not statistically different (*p* = 0.83, 0.78, 0.23, and 0.21 respectively) (Table 2). Compared to C17, rate constants for C18, C28, C30, and C32 alkanes were lower by 47, 41, 36, and 41% respectively and were statistically different (*p* ≤ 0.003). The lowest rate constant observed for C36 was only 7% of the rate constant for C17 (*p* < 0.001). In general, the rate constants of longer chain alkanes were smaller than the short chain alkanes, consistent with preferential biodegradation of short chain alkanes. Taken together, the rate constant for total alkanes was similar to the rate constant for total PAHs and they were not statistically different (*p* = 0.38).

### Comparing chemical signature of submerged oil mats, snare oil, and SRBs

A fifth sampling event, conducted immediately after TS Lee in September 2011, collected two additional categories of samples; pieces of submerged oil mat (SOM) that had been broken up by the storm and distributed on the beach, and oil associated with “snare,” a pom pom-type of oil absorbent consisting of polypropylene strands that occasionally was left on the beach and buried by sand. Biodegradation ratios for the SOM and snare samples are compared with the initial SRB samples (10/26/10) and SRB samples collected from Elmer's Island after the passage of TS Lee in 2011 in Table 3. The SOM samples had the highest measured PAHs of all the samples and the total PAH normalized to hopane was 19 ± 5.8, which is approximately twice the amount observed from 10/26/2010 sampled SRBs (Table 3) and an order of magnitude larger than snare samples and SRBs from Elmer's Island. C2-phenanthrene data had similar trends although C2-phenanthrenes were nearly completely depleted in the Elmer's Island samples. Alkanes were highest in the SOM samples and lowest in the Elmer's Island SRBs that were on the beach the longest.

**Table 3:** **Comparison of submerged oil mat (SOM) hopane ratios with SRBs from Fourchon Beach (FB) and Elmer's Island (EI)**.

**Sample**	**Date**	∑**PAH/hopane**	**C2-PHEN/hopane**	**C1-CHRYS/hopane**	∑**Alkane/hopane**
FB-SRBs	10/26/10	7.2 ± 0.84	2.2 ± 0.3	0.19 ± 0.05	130 ± 11
SOM	9/8/11	19 ± 5.8	4.8 ± 1.5	0.49 ± 0.12	199 ± 27
Oil snare	9/8/11	1.0 ± 0.17	0.11 ± 0.04	0.15 ± 0.03	40 ± 8.8
EI-SRB	10/20/11	1.7 ± 0.31	0.08 ± 0.03	0.39 ± 0.08	13 ± 9.6

When plotted on the double ratio plot (Figure 3), snare oil and SOM samples plotted near the initial SRB samples taken in October 2010 and the oil sampled immediately after the spill (Diercks et al., 2010). Based on the values from Figure 3, SOM samples are similar to the oil that first reached the shoreline, even though they were over a year old. Snare oil samples plotted similar to an unweathered sample, even though results of hopane ratios indicated a much more degraded profile. These samples are in sharp contrast to the SRBs from the supratidal, which showed consistent changes in C2/C3 phenanthrene and dibenzothiophene ratios over time (Figure 3).

### Biogeochemical parameters

Biogeochemical parameters relevant to the biodegradation of crude oil components in SRBs have been measured in 3 previous studies (OSAT-II, 2011; Urbano et al., 2013; Lemelle et al., 2014). Available chemical, physical and microbial data are summarized in Table 4. Review of those parameters identified data gaps that would improve assessments of biodegradation potential of crude oil in SRBs. These included data to compute molar C:N ratios and a more extensive set of measurements of moisture content. %C, %N and moisture content were assessed on a series of SRB and SOM samples from Fourchon Beach and Elmer's Island (Table 5).

**Table 4:** **Summary of published biogeochemical parameters of SRBs and submerged oil mat (SOM) samples**.

**Parameter**	**Value**	**References**
Porosity	0.35 (SRBs)	OSAT-II, 2011; Urbano et al., 2013
	0.07 (SOM)	
Mass	14.5 ± 3.0 g; 5.6 ± 2.3 g (SRBs from 2 locations)	Urbano et al., 2013
	31.3 ± 2.6 g (SOM)	
Moisture	<0.5% (SRBs)	Urbano et al., 2013
Projected area	106.4 ± 134 mm^2^; 55.2 ± 113 mm^2^ (SRBs from 2 locations)	Lemelle et al., 2014
% Oil	4.2–12.8% (SRBs)	OSAT-II, 2011
	9.4–16.8% (SOMs)	
N	1.4–2.0 mg/kg (SRBs) (NH^+^_4_) 7.5 mg/kg (SOM) (NH^+^_4_) 2 mg/L total N (Grand Isle beach porewater)	OSAT-II, 2011; Urbano et al., 2013
P (orthophosphate)	0.5–0.66 mg/kg (SRBs)	Urbano et al., 2013
	0.29 mg/kg (SOM)	
Salinity	1.3–1.5 ppt (SRBs) 59.8 ppt (SOM)	Urbano et al., 2013
Sulfate (water-extractable)	148–4.3 mg/kg (SRBs)	Urbano et al., 2013
	6.1 mg/kg (SOM)	
Oxygen	~220 mM (7 mg/L) after wetting 0–180 mM (0–5.76 mg/L) after 4 days in aerobic seawater 0.4 mg/L in Grand Isle, LA beach porewater	OSAT-II, 2011; Urbano et al., 2013
Microbial populations	Known PAH degraders: *Mycobacterium* sp. (SRBs)	Urbano et al., 2013

**Table 5:** **C:N ratios and moisture content in SRBs, SOM and snare oil from Fourchon Beach (FB) and Elmer's Island (EI)**.

**Sample**	**Date sampled**	**Location**	**Moisture content (%)**	**% C**	**% N**	**C/N (molar)**
**SUPRATIDAL SRBs**						
A 3-1	8/4/2011	FB	0.99	5.44	0.04	159
A 3-2	8/4/2011	FB	0.81	5.54	0.04	162
Z9-SS	3/1/2011	FB	0.35	5.54	0.02	323
B 14-12 s	5/19/2011	FB	0.35	4.78	0.03	186
EM-5	2/9/2012	EI	0.26	2.25	0.02	131
EM-18B	2/9/2012	EI	0.31	2.72	0.02	159
**SUBMERGED OIL MATS**						
MAT-1	9/16/2011	FB	1.02	14.19	0.04	414
BC-2	12/15/2011	FB	1.80	8.15	0.03	317
TB12	9/8/2011	FB	0.92	12.18	0.03	474
**INTERTIDAL SNARE OIL**						
S20	9/8/2011	FB	1.75	6.68	0.07	111

Moisture contents were all below 2% by weight and those of supratidal SRBs were all below 1%. C:N molar ratios for all samples were larger than 100:1 (Table 5). Supratidal SRB C:N ratios ranged from 131 to 323 while SOM ratios were substantially higher (317–474). Intertidal snare oil has the lowest ratio at 111, consistent with regular washing with Gulf of Mexico seawater with approximately 1 mg N/L of seawater (Urbano et al., 2013).

## Discussion

Changes in SRB PAH/hopane ratios over time (Figure 2) are consistent with a biodegradation process. Declines in hopane ratios for the three-ring PAHs (phenanthrenes and dibenzothiphenes) over time on the beach are in contrast to relatively constant 4-ring chrysene and C1-chrysene hopane ratios. When double-ratio plots are used to track relative biodegradation of the three-ring alkylated PAHs in the supratidal SRBs, a clear pattern of change is observed as C2-PHEN and C2-DIBENZ are degraded preferentially to their C3 alkylated homologues. The extent of MC252 oil components losses observed in Fourchon Beach and Elmer's Island SRBs over 19 months are similar to those observed after 8 years in the Exxon Valdez spill (Michel and Hayes, 1999). Also, the profiles of MC252 oil components observed on Fourchon Beach and Elmer's Island are similar to those observed in MC252 oil analyzed from Pensacola, FL beaches (Kostka et al., 2011) and Marsh Point MS marsh samples (Liu et al., 2012). These changes include loss of alkanes <C15 and the alkylated naphthalenes. Patterns of change were similar to those of a biodegradation process not a physical weathering process (Wang et al., 1998), namely the decline of lower alkylated homologues and persistence of higher molecular weight PAHs. Within these SRBs, however, oxygenated residues representing partial biodegradation products of crude oil components were present in significant concentrations (Aeppli et al., 2012) although PAHs may not necessarily be precursors to these byproducts (Hall et al., 2013). Disappearance of the parent PAHs may not result in complete mineralization to CO_2_.

For alkanes, consistent declines in hopane ratios were also observed in SRBs on the beach over time (Figure 4). Initial ratios of C26 to lower molecular weight alkanes (C15–17) were 1–2 orders of magnitude higher than concentrations at sea, suggesting that substantial biodegradation of these alkanes had already occurred prior to our first sampling event on shore. On the beach, ratios declined consistently over time as C26 continued to degrade relative to the low concentrations of C15–C17 remaining in the aggregates. Nevertheless, concentrations of higher molecular weight alkanes persisted in the SRBs, even until the final sampling date. Rate constants, measured from losses of alkanes and PAHs in the SRBs, were of the same order of magnitude as those measured during an experimental oil spill on Delaware Bay, USA (Venosa et al., 1996). Rate constant of PAH loss from the Delaware Bay study were approximately double those of the rates of PAHs measured in SRBs from Fourchon Beach while alkane rates from control treatments were approximately 3 times rates observed in the SRBs.

While biodegradation ratios, relative patterns of PAH and alkane loss and computed rate constants suggests that biodegradation reactions are occurring, is there evidence that hydrocarbon-degrading microbial populations can colonize these aggregates? Previous measurements of eubacterial and sulfate-reducing microbial populations within several SRBs using DGGE revealed several trends (Urbano et al., 2013). SRB microbial populations differed with position on the beach, with populations within supratidal SRBs distinct from intertidal snare oil and SOM samples (Urbano et al., 2013). Specifically, known PAH degrading genera such as *Mycobacterium* (Khan et al., 2002; Uyttebroek et al., 2006) and *Stenotrophomonas* (Juhasz et al., 2000; Papizadeh et al., 2011) comprised significant bands in the supratidal and intertidal snare samples, respectively (Urbano et al., 2013). These results compliment microbial community structure analyses in oiled beach sands in Florida (Kostka et al., 2011) that revealed a diverse response, including sequences derived from members of oil-degrading taxa such as *Gammaproteobacteria* (*Alcanivorax, Marinobacter*) and *Alphaproteobacteria* (*Rhodobacteraceae*).

Biogeochemical conditions present in SRBs need to be examined critically to develop hypotheses about the rate and extent of observed concentration changes. These may shed insight to data trends including the detection of alkanes in SRBs after over a year and a half on the beach, the persistence of PAHs and alkanes in SOM samples and the relatively high variability in analyses between SRBs, particularly for alkanes. From a review of previously collected data and the values measured in the current study, four biogeochemical parameters are of concern as potentially limiting biodegradation rate and extent. These are moisture content, oxygen concentrations, N concentrations and salinity.

Moisture content of SRBs are very low (<1%) on the supratidal beach environment in periods between rainfall events (Urbano et al., 2013). Additional measurements (Table 5) were conducted for this study and were also very low, ranging from 0.26–1.8%. Supratidal SRBs had lower moisture contents when sampled when compared with SOM or snare oil samples that were regularly inundated (Table 5). SRBs appear to have poor water holding capacities and rely on regular rainfall or tidal inundation to replenish moisture. These sampled water contents are well below concentrations optimal for biodegradation (Dibble and Bartha, 1979; Hejazi and Husain, 2004; Tibbett et al., 2011). These studies maintained water concentrations between 30 and 80% of field capacity, and between 50 and 70% of field capacity for optimal results. For SRBs, measured moisture contents would represent field capacities of between 2 and 9% given our knowledge of porosity, density and volume of typical SRBs. Given the rainfall patterns on the beach and the frequency of storm-driven tides, infrequent periods of wetting would be interspersed with longer drying periods leading to suboptimal moisture concentrations. *Mycobacterium*, observed as one of the prominent bands in the supratidal SRBs, can be adapted to low moisture content and periods of dessication (Wick et al., 2003; Harland et al., 2008) and this capability may have insured that biodegradation of PAHs continued between rain events on the beach.

*In situ* oxygen measurements are not available in the very limited water in field-sampled SRBs but microelectrode measurements from SOM pieces incubated in the laboratory (Urbano et al., 2013) show that O_2_ is at saturation in aggregate porewater immediately after wetting. As the SOM is submerged, however, O_2_ is consumed over a several day period to produce a large zone of anaerobiosis within the aggregate. While these measurements are limited, the level of carbon observed in the aggregates, the presence of aerobic hydrocarbon degraders and the porosities observed supports the development of anaerobic conditions. This can help explain relative PAH and alkane persistence in SOM samples. SOM samples had higher alkane/hopane and PAH/hopane ratios than the initial SRB samples, despite sampling a year later. Because of their position on the beach, these oil forms were consistently submerged and likely anaerobic over the course of their time in the system. While alkanes have been observed to degrade under sulfate-reducing conditions (Caldwell et al., 1998; Townsend et al., 2003), alkane concentrations still persist in these SOM samples. In contrast, the continued biodegradation of these compounds in the SRBs on the supratidal is likely greatly enhanced by the regular wetting by aerobic rainwater followed by drying which limits the formation of anaerobic conditions within the aggregates. *Mycobacterium sp*., observed in the supratidal SRBs only, is capable of degrading pyrene at O_2_ concentrations as low as 3 μM (Fritzsche, 1994), which may allow them to function in that critical time after rewetting when oxygen is being consumed.

Hydrocarbon biodegradation requires a source of nutrients and can be commonly limited by N and P concentrations lower than optimal levels (Dibble and Bartha, 1979). Previous measurements of exchangeable NH^+^_4_ and soluble NO^−^_2_/NO^−^_3_ in SRBs demonstrated 3 features of N chemistry in the aggregates (Urbano et al., 2013). First, exchangeable ammonium was the dominant labile form of N in the aggregates ranging from 1.39 to 12.6 mg of exchangeable ammonium per kg of SRB. Second, concentrations of N varied with position on the beach with SRBs in the intertidal possessing higher concentrations of N. This is consistent with regular inundation with seawater that contains about 1 mg/L of N at this location. Third, SRBs that were present on the supratidal the longest period of time had the lowest concentrations of N. These data suggest that nutrient content is due to the position of the SRBs on the beach but the form of the nutrients within the aggregate can be influenced by microbial activity.

The previous data (Urbano et al., 2013) does not allow easy computation of absolute amounts of N relative to the carbon, primarily crude oil, present in the aggregates. Therefore, additional SRBs from defined classes (i.e., supratidal SRBs, SOM and snare oil samples) were analyzed for total C and N to make that comparison (Table 5). C/N molar ratios, commonly used as a diagnostic variable for hydrocarbon biodegradability, ranged from 111 to 474 in SRBs, SOM and snare oil samples. Molar C/N ratios were well-above optimal values reported for experimental aerobic hydrocarbon biodegradation in crude oil contaminated soils that were approximately 60:1 (Dibble and Bartha, 1979). Is N limiting biodegradation in the SRBs? If we consider literature values for specific PAH degrading populations, optimal molar C:N ratios are much lower. For phenanthrene degradation by *Sphingomonas* in mangrove soils, C:N ratios of 100:1 were optimal (Chen et al., 2008). For 2 important PAH degraders, *Sphingomonas* and *Mycobacterium*, soil slurry experiments showed that biodegradation can proceed under lower nutrient concentrations, C:N of 100:1, even though 100:10 were optimal (Leys et al., 2005). In the SRBs sampled here, C:N ratios are generally 3× higher than those studies. N replenishment is occurring through several mechanisms including deposition of sea spray aerosols (Zhu et al., 2013) and occasional inundation with seawater containing N during storm events. For extended periods, though, SRBs on the beach have whatever N was retained in the aggregate.

Measurements of salinities ranged from hypersaline conditions in intertidal aggregates to low salinities in supratidal aggregates. Since salinity can be an important inhibitor for PAH consortia (Kastner et al., 1998; Diaz et al., 2002), the apparent ability of rainfall to reduce salinity to low levels may be important in sustaining biodegradation reactions in supratidal SRBs. PAH biodegradation is impacted by salinity and lower salinities (<15 ppt) are often optimal (Chen et al., 2010). A *Mycobacterium* and *Sphingomonas* culture could not grow at salinities above 19 ppt (Leys et al., 2005). SRBs on the supratidal beach environment have low salinities, presumably via washing by rainwater. In contrast, high salinities including hypersaline conditions in the SOM samples are likely contributing to the persistence of these oil forms.

Based on a critical examination of biogeochemical parameters in SRBs and SOM samples, 2 regimes on the beach can be hypothesized with respect to biodegradation; a moisture and nutrient-limited regime on the supratidal and an oxygen and salinity-limited regime in the intertidal. Despite these challenges, biodegradation processes in SRBs occurs at rates and timeframes that are relevant to the coastal transport processes that move them off the beach and into the adjacent mudflats and marshes. In contrast, SOM samples do not appear to be amenable to biodegradation while in the intertidal environment where oxygen limitations and salinity impacts may be inhibitory. Intact mats also have much lower surface areas relative to the SRBs which may contribute to persistence as well. As these mats break up and are deposited on the beach, they represent new oiling very similar to the chemical quality of oil that reached the beach immediately after the spill began. Finally, while PAH and alkanes represent significant components of MC252 that have both aerobic and anaerobic biodegradation potential, it should be emphasized that many other classes of compounds exist in crude oil whose behavior may not be accurately represented by examining these 2 groups alone. The overall decline in C content in the aggregates (Table 5) is evidence, however, that additional components of the mixture are degrading.

While the chemical evidence can be explained by biodegradation, what about the other potential weathering processes such as evaporation, dissolution and photo-oxidation? SRB samples were formed from highly weathered oil and significant evaporative (Middlebrook et al., 2012) and dissolution (Reddy et al., 2012) losses had occurred in the water column and on slicks prior to reaching the shoreline. Alkanes less than C15 and lower molecular weight PAHs such as alkylated naphthalenes were essentially absent from the SRBs analyzed in this study. The remaining components have lower vapor pressures and lower solubilities and therefore, are less susceptible to these weathering processes. SRB structure and position on the beach creates mass transfer limitations in the aggregates for these processes. Dissolution of oil components in the aggregates is limited by infrequent wetting events while evaporation is limited by layers of cleaner sand adhering to the oilier core. This structure creates similar limitations for continued photooxidation within SRBs even though this process was likely important at sea (Aeppli et al., 2012; Genuino et al., 2012). Taken together, these data provide a weight of evidence that biodegradation is occurring but not conclusive proof. Only controlled studies that include killed controls can confirm unequivocally that a microbial process is responsible for chemical changes within these unique oil:sand aggregates.

## Author contributions

Vijaikrishnah Elango wrote the first draft of the manuscript and provided day-to-day supervision of study objectives. Marilany Urbano performed biogeochemical analyses, chemical analysis of SOM samples and reviewed biogeochemical data. Kendall R. Lemelle performed chemical analysis of SRB samples. John H. Pardue designed studies, provided oversight and edited final draft of manuscript.

### Conflict of interest statement

The authors declare that the research was conducted in the absence of any commercial or financial relationships that could be construed as a potential conflict of interest.
